# Intra-uterine packing: an effective and affordable tool in the management of post-partum hemorrhage - cohort study

**DOI:** 10.11604/pamj.2023.45.192.39441

**Published:** 2023-08-31

**Authors:** Ahmed Halouani, Yassine Masmoudi, Aymen Hammami, Hafedh Abbassi, Amel Triki, Anissa Ben Amor

**Affiliations:** 1 Gynecology and Obstetrics Department, University Hospital Mongi Slim Hospital la Marsa, Tunis, Tunisia,; 2 Faculty of Medicine of Tunis, University Tunis El Manar, Tunis, Tunisia

**Keywords:** Conclusion: intra uterine packing is a valuable tool in managing postpartum hemorrhage due to uterine atony

## Abstract

**Introduction:**

postpartum hemorrhage is the main cause of maternal death worldwide. Uterine balloon packing has shown promising outcomes in PPH management. Nevertheless, its usage is limited in low- and middle-income countries due to associated costs. Uterine packing using gauzes presents a potentially efficient and cost-effective alternative. This study aims to assess the safety and efficacy of intra-uterine packing with gauzes in managing postpartum hemorrhage.

**Methods:**

this was a retrospective study over a period of two years and six months. All patients who experienced PPH due to uterine atony during vaginal delivery, with no response to medical first-line treatment, were included. IUP using gauze was employed as a second-line intervention. The primary outcome was the success of postpartum hemorrhage management. Secondary outcomes included patient vitals, the need for blood transfusion, change in hemoglobin levels (delta Hemoglobin), and maternal morbidity (post-partum infection, Sheehan syndrome, and retained gauzes).

**Results:**

the study included 63 patients. The mean age was 30.06 ± 5.6, the mean gravida was 2.65 ± 1.9 and the mean para was 2.12 ± 1.31. None of these patients experienced major complications following gauze insertion. Three patients underwent laparotomy and conservative surgical management was performed. Hysterectomy was not required for any participant, and no maternal deaths were recorded.

## Introduction

Postpartum hemorrhage (PPH) is a leading cause of direct maternal mortality worldwide, accounting for approximately one-quarter of all maternal deaths [1]. The current standard second-line intervention involves intra-uterine packing (IUP) using a Bakri balloon [2]. However, this approach faces limitations in low- and middle-income countries due to its associated expenses [3]. As an alternative, IUP with gauzes emerges as an interesting and potentially cost-effective alternative.

The use of IUP with gauze is supported by limited evidence. Recent studies have demonstrated promising outcomes in managing PPH, with a high success rate and no adverse effects on fertility [3]. In our region, the intermittent unavailability of Sulprostone and Bakri balloon has led to the need for integrating IUP with gauzes as an additional intervention within the PPH management protocol. This decision is driven by the potential benefits and its capacity to prevent the need for more invasive surgical measures. The aim of this study was to assess the effectiveness of the IUP with gauzes in the PPH management.

## Methods

**Study design and setting:** this was a retrospective observational study over a period of two years and six months from January 2020 to June 2022. This study was conducted at the department of Obstetrics and Gynecology of the University Hospital Mongi-Slim, La Marsa, Tunis, Tunisia.

**Study population:** patients who experienced PPH due to uterine atony during vaginal delivery and did not respond to first-line medical interventions were enrolled in the study. Patients with perineal and cervical lacerations, placental adhesion abnormalities, and those who were hemodynamically unstable were excluded.

**Figure 1:**
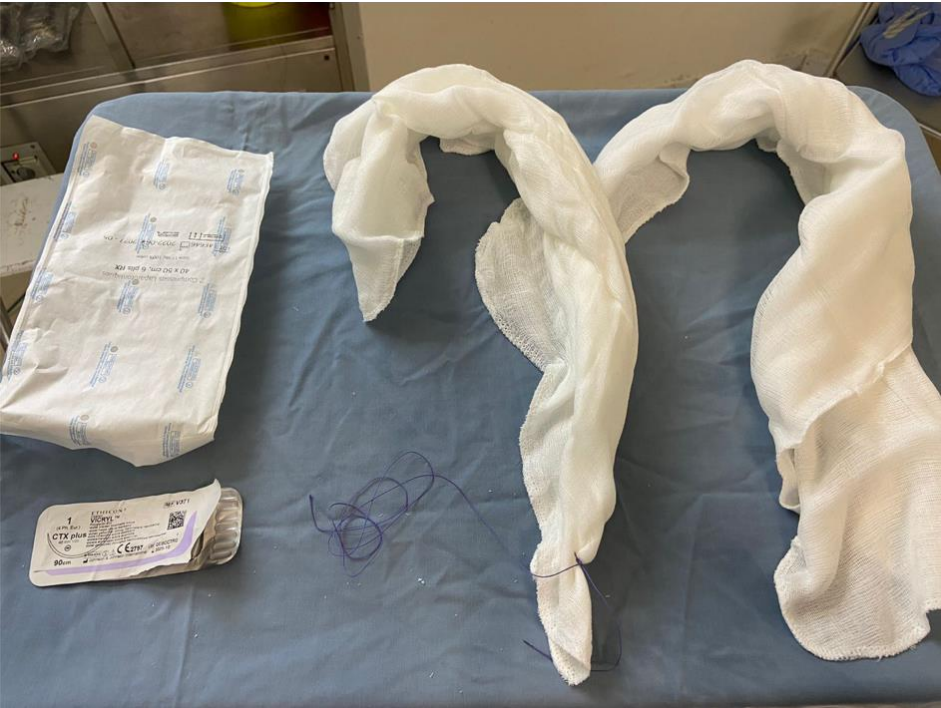
gauze attached with suture thread

**Figure 2:**
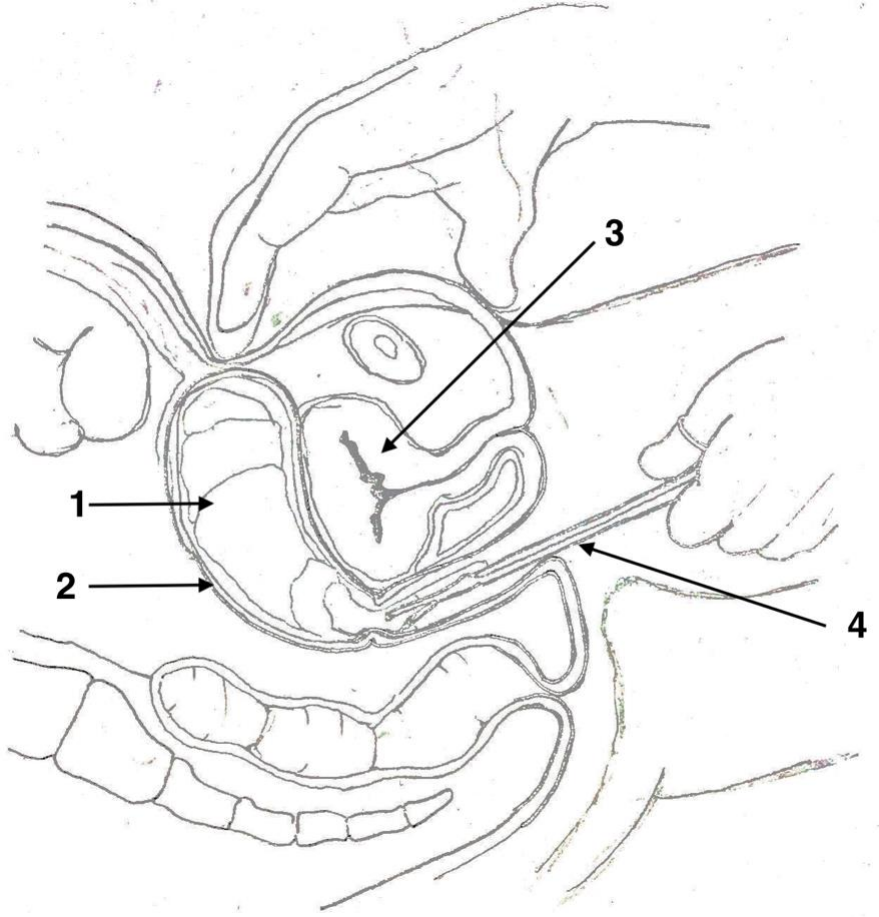
intra-uterine packing inserted manually (1: gauze, 2: uterus, 3: bladder, 4: dressing forceps)

**Study protocol:** post-partum hemorrhage was defined as a blood loss greater than 500 mL following a vaginal delivery [4]. Blood loss was measured using a bag collector. PPH management was based on a protocol that included the medical treatment as a first-line intervention using oxytocin followed by Sulprostone (Or Misoprostol) and tranexamic acid. In cases where the first-line medical treatments were unsuccessful, uni-meshes cotton hydrophile gauzes (32 x 36 cm dimensions) were used for packing (Figure 1). The IUP with gauzes was performed under ultrasound guidance. The threads were attached to it to facilitate its removal (Figure 2). The gauzes were removed within 24 hours by gentle traction on the thread. Antibiotic prophylaxis of 3 days using a large spectrum antibiotic was administrated.

**Study outcomes:** the primary outcome of the study was determined by the success IUP, defined as the total arrest of bleeding following the procedure. The procedure was deemed a failure if bleeding persisted, worsened, or if the patient's condition became hemodynamically unstable after the packing. In such cases, the gauzes were immediately removed, and surgical interventions were initiated. The secondary outcomes were the patient's vitals, the need for blood transfusion, delta hemoglobin, and maternal morbidity (post-partum infection, Sheehan syndrome, and omitted gauzes).

**Data collection:** clinical characteristics, laboratory findings and treatment procedures were retrieved from medical record (obstetrics and anesthesia files).

**Statical analysis:** results were expressed on means and standard deviations.

**Ethical considerations:** the study protocol was approved by the ethical committee of Mongi-Slim University Hospital, La Marsa, Tunisia (approval no. 23/2022) on August 4^th^, 2022. All Women gave their written consent to take part in the study.

## Results

**Figure 3:**
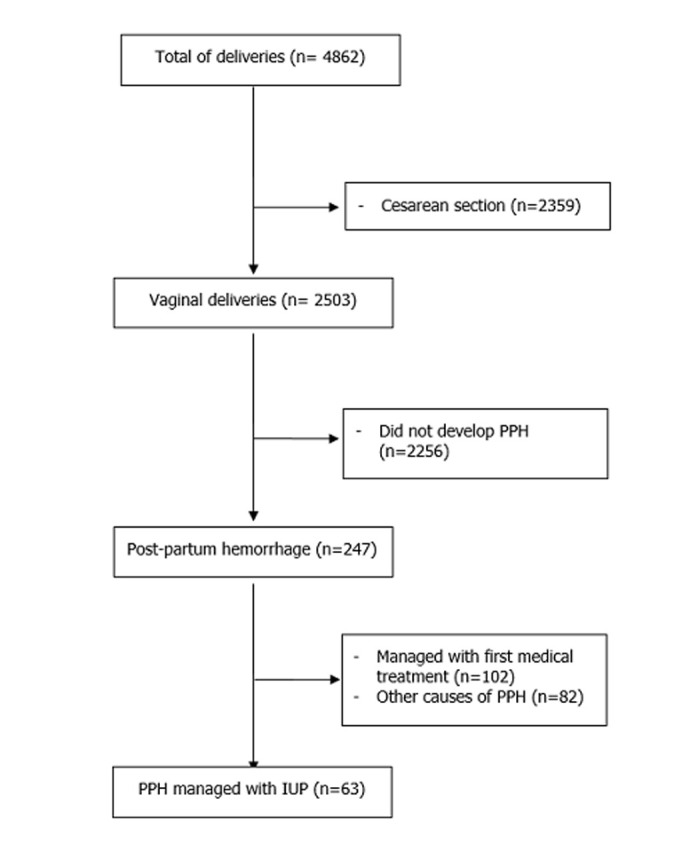
flowchart of intrauterine packing

**Participants:** a total of 4862 women delivered between January 2020 and June 2022 in the maternity ward of Mongi Slim La Marsa, Tunis, Tunisia. Among the 2503 women who delivered vaginally, 247 (9.86%) developed PPH. A total of 184 (74.5%) patients were excluded as the PPH was either managed successfully with the first line medical intervention or occurred due to another cause than uterine atony or in cases where the patient was clinically unstable. Intrauterine packing was performed in 63 patients (25.5%) (Figure 3).

**Descriptive data:** risk factors for PPH were noted in 42 patients (67%). These risk factors included an overdistended uterus due to a large gestational age fetus or in case of twin pregnancy, multiparity, induction of labor, fast or long labor, and chorioamnionitis (Table 1). The average birth weight was 3432 ± 428 g. In ten cases, birth weight exceeded 4000g (16%).

**Table 1:** baseline characteristics and IUP outcomes

Characteristics	Results
**Maternal age, y ± sd**	30,06 ± 5,6 (18-45)
**Body mass index, kg/m**	30,55
**Gestational age, wk ± sd**	39,7 ± 7,4
**Parity**	
**Nulliparous, %**	30
**Parity >2, %**	70
**Birthweight, g ± sd**	**3432 ± 428**
**Risk factor of PPH, %**	
**None, %**	33
**Macrosomia, %**	20
**Twins or triplet, %**	6
**Inducted labor, %**	16
**Rapid labor, %**	9
**Hight parity (>3), %**	12
**Chorioamnionitis, %**	4
**Delta hemoglobin ± sd**	5,24 ± 3.8
**Number of red cells units (min-max)**	0,8 (0-6)
**Number of fresh frozen plasma unit (min-max)**	0,9 (0-8)
**Surgical procedure, %**	
**B-Lynch, %**	4
**Uterine artery ligation, %**	3
**Hysterectomy, %**	0
**Intensive care unit, %**	19
**Antibiotics, %**	100
**Postoperative CRP (> 6 mg/l), %**	14

**IUP:** Intra-uterine packing; **PPH:** Psost-partum hemorrhage; **CRP:** C-Reactive Protein; y ± sd: Represents Years ± standard deviation; wk ± sd: Denotes week ± standard deviation; g ± sd: Signifies gramme ± standard deviation.

**Outcome data:** intra-uterine packing was performed in 42 cases (67%) by a specialty trainee and in 21 cases (33%) by a consultant obstetrician. None of these patients suffered from a major complication that could be linked directly to the use of the gauze. At least three gauzes were used for each patient. In case of over-distended, more than 3 gauzes were needed. The mean drop of hemoglobin level (delta hemoglobin) was 5.24 g/dl ± 3.8. Seventeen (27 %) patients needed at least 2 units of red blood cells with a maximum of 6 administrated. Nine patients (14 %) presented postoperatively with fever associated with high CRP. Three of them were diagnosed with chorioamnionitis (4%) and were successfully managed with large-spectrum antibiotics administrated intravenously at first then switched to oral for a total of 14 days. No patient developed a septic choc and no admission to the intensive care unit or infectious disease ward was needed. In one case, gauze was left inside after the patient´s discharge and had to be removed seven days later.

**Main result:** during the period of the study (30 months), no lifesaving hysterectomy was needed. In only 3 cases (4%), laparotomy had to be performed for conservative surgical measures including Tsirulnikov ligation, B-Lynch uterine sutures and internal iliac artery ligation. Twelve (19 %) patients were admitted to the intensive care unit and only one patient developed Sheehan syndrome. The mean delta hemoglobin was 5.24 g/dl ± 3.8. Among all patients included, 3 patients had a subsequent pregnancy. In one case a gauze was left in the uterus and had to be removed 7 days later.

## Discussion

The aim of the study was to assess the safety and efficiency of IUP with gauzes in the second-line management of PPH. In this study, 63 patients experienced severe PPH and were treated with IUP with gauzes. The success rate was 95%, with only three patients requiring a laparotomy for conservative surgical interventions. No maternal death was recorded. The mean delta hemoglobin was 5.24 g/dl ± 3.8. Twelve patients were admitted to the intensive care and they experienced favorable outcomes. Currently, the IUP with a balloon is the validated second-line intervention for managing PPH. In a meta-analysis conducted by Suarez *et al*. the effectiveness and safety of uterine balloon packing were evaluated [5]. The authors reported an overall success rate of approximately 85.9% (95% confidence interval [CI], 83.9-87.9%), with the highest success rates observed in cases of PPH caused by uterine atony (87.1%; 95% CI, 84.1-89.9%). Nevertheless, the implementation of this device faces challenges in low-resource countries due mainly to its cost. The lack of the Bakri balloon is a crucial situation that increases rates of morbidity and mortality associated with PPH.

It is essential to explore alternative interventions that demonstrate similar outcomes in managing PPH, while also being readily available and cost-effective. Intrauterine packing with gauze appears promising as it replicates the tamponade effect of the balloon at a lower cost (2USD) and with greater accessibility. Our study found a success rate of approximately 95% with the IUP with gauzes. However, further confirmation of these results is necessary through a large randomized controlled trial comparing IUP with gauzes to IUP with the Bakri balloon for the management of PPH. Such evidence can provide valuable insights for clinical practice and improve maternal health outcomes.

IUP with gauze is based on low-level evidence, this is due to the lack of studies that evaluate this tool. One of the rare authors who worked on this subject was Makosso *et al*. They conducted a retrospective study to evaluate the efficacy of the IUP in the management of PPH [6]. The authors report 99 patients with PPH who were managed with IUP with gauzes. The success rate was comparable to our study, at 91.9%, and the number of patients experiencing a failure of IUP with gauzes was limited to only eight. Three patients required laparotomy and underwent conservative surgery, while the remaining five patients underwent hysterectomy. The PPH management protocol of the American College of Obstetricians and Gynecologists (ACOG) recommends the utilization of gauzes soaked with thrombin [4]. Additionally, the literature describes the use of chitosan-covered gauzes, which have shown comparable results to IUP with a balloon in the management of PPH. The study revealed no statistically significant difference between the two groups concerning patients' vital signs (pulse, blood pressure, and temperature), hemoglobin levels, estimated blood loss, and admission to the intensive care unit. The study underlines also the lower price of the gauze compared to the Bakri Balloon [7].

The extraction of gauzes can be easily accomplished by gentle traction on the gauze thread. however, inadvertent retention of gauze may occur. In our study, we observed one case where a gauze was unintentionally left in the uterus and required removal after seven days. A similar complication was reported by C. Schmid *et al*., they involved the use of chitosan-covered gauze for the management of PPH, leading to persistent spotting postoperatively due to residual gauze [8]. To prevent such complications, it is crucial to document the number of gauzes used for each patient in the operative report. However, considering the stress of managing PPH, there is a risk of overlooking counting items. Therefore, based on our experience, we recommend performing an ultrasound after gauze removal to confirm uterine vacuity.

The main strength of our study was the number of patients enrolled. However, the study has some limitations, primarily due to its retrospective design and single-center setting, the absence of patient satisfaction and tolerance assessment during the gauze removal process. Moreover, long-term outcomes regarding future fertility remain a subject of interest for further investigation.

## Conclusion

Intrauterine packing with gauzes is an efficient tool in the management of PPH with a success rate of 95%. This tool can be considered in the second-line management of PPH in the absence of Bakri Balloon.

### What is known about this topic

- *post-partum hemorrhage is the first leading cause of maternal death;*
- *Intra-uterine packing with is a validated tool in the management of post-partum hemorrhage;*
- *Intra-uterine packing with gauze efficiency is based on low-level evidence*.

### What this study adds

- *Intra-uterine packing with gauze is a low-cost tool for the management of post-partum hemorrhage;*
- *Intra-uterine packing with gauzes seems to be an effective alternative in the management of post-partum hemorrhage;*
- *Intra-uterine packing with gauzes can be associated with some specific side effect (gauze omission)*.
